# Pitch at the Cocktail Party: A Comparative Approach to Studying Selective Attention

**DOI:** 10.3390/biology15080618

**Published:** 2026-04-15

**Authors:** Joel Ward, Veronica M. Tarka, Artem Diuba, Kerry M. M. Walker

**Affiliations:** 1 Department of Physiology, Anatomy & Genetics, University of Oxford, Oxford OX1 3PT, UK; joel.ward@nds.ox.ac.uk (J.W.); veronica.tarka@dpag.ox.ac.uk (V.M.T.); artem.diuba@dpag.ox.ac.uk (A.D.); 2 Nuffield Department of Surgical Sciences, University of Oxford, Oxford OX3 9DU, UK

**Keywords:** pitch, attention, hearing, auditory cortex, harmonics, listening

## Abstract

Pitch is a powerful cue for segregating sound sources in complex acoustic scenes, yet the neural mechanisms through which it guides selective attention remain unclear. In this review, we consider behavioural and neurophysiological evidence from humans and animal models to examine how pitch supports selective listening in a two-stage process: bottom-up pitch-based feature binding, followed by top-down enhancement of an attended sound source. Behavioural studies demonstrate that even modest pitch differences substantially improve listeners’ segregation of harmonic sounds, tone streams, and competing talkers. Human EEG, MEG, fMRI and ECoG studies show enhancement of target sound representations in auditory cortex during selective listening, but understanding this process at the level of individual neurons requires further study in animals that are trained in pitch-based selective listening tasks. Other key questions in this field include the relative roles of resolved and unresolved harmonic cues, the neural circuit mechanisms underlying target enhancement versus masker suppression, and how attention can target distributed cortical pitch representations. We argue that cross-species, naturalistic paradigms are essential for answering these questions and for addressing the listening difficulties associated with ageing and hearing loss.

## Simple Summary

Natural environments often contain overlapping sound waves from many different sound sources. For example, to follow a conversation in a crowded restaurant, the brain must separate these sounds so we can focus on our friend’s voice. Pitch is the tonal quality that makes a sound seem low or high, and it plays a critical role in guiding our attention. However, the brain mechanisms that support this process remain poorly understood and understudied. Here, we describe how improved behavioural tasks and neural recording studies in both humans and animal models could help advance our knowledge of how pitch helps us listen in complex environments.

## 1. Introduction

Following a conversation in a noisy restaurant is a familiar challenge, and one that becomes especially difficult with ageing or hearing loss. Understanding how the brain solves this “*cocktail party problem*” is a central aim of auditory neuroscience, and it is commonly conceptualised as a two-stage process [1]. In the first *feature binding* stage, the ascending auditory pathway extracts complex acoustic features, such as harmonic structure, temporal synchrony, and spatial location, and binds these into perceptual objects [2,3,4]. In the second stage, *top-down attention* selects and enhances target sound representations in the auditory system [5,6]. Pitch is among the most potent cues for sound segregation [2,7], yet how it guides attention at the level of individual neurons and microcircuits remains poorly understood.

Pitch, typically defined as the tonal quality of a sound along a low-to-high scale [8], is a salient perceptual feature of speech and other harmonic sounds. Acoustically, the pitch we hear corresponds to the sound’s fundamental frequency (F0). A sound that evokes a pitch is typically periodic at F0, and all its frequency components (the *harmonics*) are integer multiples of F0.

Animals, like humans, can detect, discriminate, and order pitch [9,10,11,12,13], and neural representations of pitch have been described in the auditory cortex of many mammalian species [14]. Attention to task-relevant sound sources modulates auditory cortical responses in both humans [6,15] and non-human animals [5,16,17]. Studies on pitch-based selective listening support the translation of circuit-level measurements and neural manipulations that are only feasible in animal models to cognitive and language processes that are unique to humans. Integrating human and animal research is therefore essential to uncovering the neural mechanisms for pitch-based selective listening.

Longstanding insights into selective listening have come from experiments that used pure tones or harmonic tone complexes [1,18,19,20]. Determining the frequency of a pure tone is mechanistically trivial for the auditory system, as it is represented as the place of excitation on the tonotopic map, and does not require F0 extraction across multiple harmonics. While these simple stimuli isolate acoustical cues well, they do not capture the complexity of overlapping voices, fluctuating noise, and dynamic spectral-temporal patterns that characterise natural scenes. More ecologically relevant paradigms use speech, vocalisations, and other environmental sounds, which have highly complex neural representations [6,21,22,23,24], and may better probe how binding and attention operate in realistic contexts [23]. Moving beyond pure-tone stimuli is critical to translate findings into clinical applications, as most people with hearing loss struggle to follow conversation in noisy environments, even if their pure tone audiogram is only mildly impaired [25,26].

This perspective review synthesises behavioural and neurophysiological evidence in humans and animal models on how pitch perception guides selective listening, particularly in the context of verbal communication. We highlight gaps in our current understanding, and propose how the open questions in this field can be addressed by methodological innovations and cross-species comparisons.

## 2. Behavioural Evidence for Pitch-Based Selective Listening

**Figure 1:**
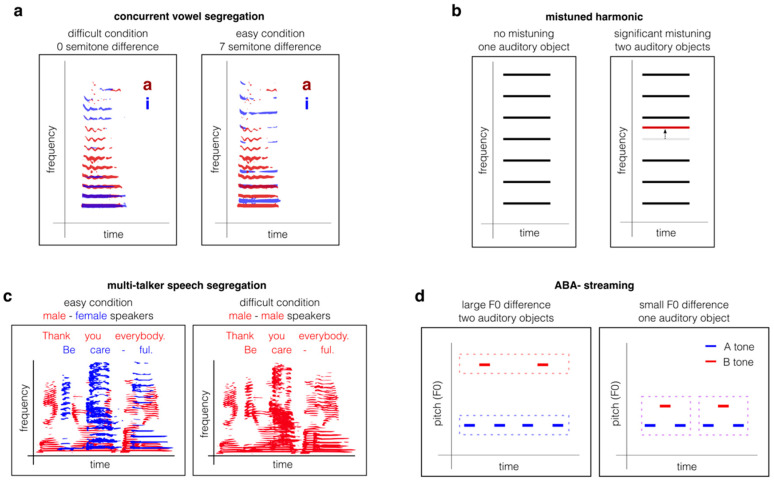
**Common paradigms for measuring pitch-based feature binding and selective attention.** (**a**) Concurrent vowel segregation tasks the listener with identifying two simultaneously presented vowels that differ only in F0. As pitch separation increases between the vowels, in this case /a/ (red) and /i/ (blue), the vowels become easier to identify. (**b**) When one harmonic within a tone complex is sufficiently mistuned (red), it is no longer a perfect multiple of the sound’s F0. The mistuned harmonic ‘pops out’ to form a separate auditory object with a different perceived pitch from the other perceptually fused harmonics. (**c**) Multi-talker speech segregation tasks present the listener with sentences spoken by two people simultaneously. The listener must identify words spoken by the target talker, who is identifiable by some feature such as gender, relative onset timing, or the first word spoken. This task becomes easier when there is a pitch separation between the talkers, typically provided by one talker being male (red) and the other female (blue). (**d**) ABA- streaming (also known as two-tone streaming) paradigms present two interleaved sequences of tones at different rates. The sequences of A (blue) and B (red) tones are perceived as a single stream of tone triplets with a ‘galloping’ rhythm when the pitch separation between the tones is sufficiently small. When the pitch separation is large, the A and B tones form two distinct perceptual streams. Lists of studies employing these 4 paradigms are provided in Table 1.

Pitch facilitates the segregation of voices in everyday listening. Listeners can more readily follow a female voice against a male voice background and vice versa, due to their pitch differences [27,28]. In fact, when talkers differed in F0 by about 100 Hz, listeners showed an intelligibility improvement equivalent to a 6 dB gain in signal-to-noise ratio [27]. Even modest pitch differences of 1–4 semitones can improve identification of two simultaneously presented vowels [29,30,31,32] (Figure 1a). Thus, pitch may play a particularly important role in segregating voices that overlap in location and time.

*Harmonicity* refers to the extent to which the frequency components of a sound are integer multiples of F0, and this acoustical correlate of pitch may play a particularly important role in binding. Mistuning a single harmonic in a tone complex by as little as 1–3% can cause it to be heard as a separate auditory object, demonstrating the strength of harmonic fusion [33,34,35,36] (Figure 1b). Harmonicity improves detection and discrimination of speech presented in noise [37] or with a competing talker [38] (Figure 1c). At least some of these segregation effects can be explained by the equal spacing of frequency components (*spectral regularity*) in harmonic sounds, even when those components are not multiples of a common F0 [36,39]. Nevertheless, harmonicity is especially important for pitch judgements made in noisy backgrounds [40], suggesting that we bind an auditory object’s harmonic components in order to segregate it from other sound sources.

Classic two-tone streaming paradigms have used alternating low (A) and high (B) frequency pure tone sequences to probe the use of frequency for auditory feature binding (Figure 1d). In these tasks, tones are often presented in a repeating ABA- sequence, where the hyphen indicates a silent gap after each tone triplet. Small frequency differences (<10%) between A and B produce a fused percept of a single auditory object with a “galloping” rhythm, and larger differences are perceived as two segregated A and B streams [1]. These effects generalise to harmonic complexes, where both F0 and the spectral envelope independently contribute to grouping [41,42]. Streaming paradigms allow the effects of pitch on sound segregation to be quantified through parametric control of F0 differences. However, their reliance on subjective reports of “one versus two streams” is a challenge for implementing these tasks in animal models.

Few studies have directly trained pitch-based selective listening tasks in animals, but there is evidence that harmonicity helps them to perceptually bind natural sounds. For example, animals can detect a mistuned harmonic in a tone complex [43,44,45,46]. Adapting two-tone streaming paradigms for animals offers a promising comparative approach [47]. Rather than relying on subjective reports of one or two streams, these paradigms in animals instead measure objective behavioural performance on a task that requires effective stream segregation. For example, if A and B are segregated, human listeners show improved recognition of melodies in the A stream [48]. Izumi [49] took this approach, training macaques to detect frequency contours in a target tone stream while ignoring similar contours in a distractor stream. Ma et al. [50] trained a similar two-tone streaming task in ferrets. In both cases, the animals performed better when the A and B streams had larger frequency differences, mirroring the effects observed in human listeners. Another approach to training ABA- streaming tasks in non-humans is to first train animals to discriminate between galloping and isochronous rhythms in a single A stream, and then test them on a variety of ABA- streams. This approach has been successfully used to demonstrate frequency-dependent behaviour in songbirds [51] and even goldfish [52]. These tasks enable direct cross-species comparisons of frequency separation effects on selective listening and are feasible to train in a variety of animal models. However, they are a less direct demonstration of perceptual streaming than their human counterparts. A limitation of the corpus of streaming studies in animals to date is that they have focused on pure tone streaming, so they have not tested the role of F0 in segregating spectrally overlapping sounds.

Studying selective listening with more naturalistic tasks, particularly those involving speech, remains challenging in animals. Geissler and Ehret altered the onsets of individual harmonics in a mouse pup call relative to the fundamental frequency and showed that nursing mothers were less likely to respond to these altered calls [53]. This behavioural effect could reflect either a difficulty in segregating the call from background noise or a more general impairment of call identification. Psychophysical designs in animals, where verbal instructions are not possible, must be careful to prevent alternative listening strategies. For example, animals may detect a brief spectrotemporal cue within a stream rather than sustaining attention to the target. These issues can be mitigated by including catch trials to measure how well animals sustain focus [54,55], or by using task-switching paradigms [56]. These approaches are well-suited to capture aspects of dynamic attentional control akin to the human cocktail party scenario.

In summary, pitch plays a key role in binding and selective attention in humans, as demonstrated across a range of psychophysical paradigms. Appropriate behavioural tools already exist to more extensively examine this process in animal models, but such studies must move beyond pure tone stimuli to explore the role of complex pitch in directing attention.

## 3. The Roles of Resolved Harmonics and Temporal Pitch Cues in Selective Listening

**Figure 2:**
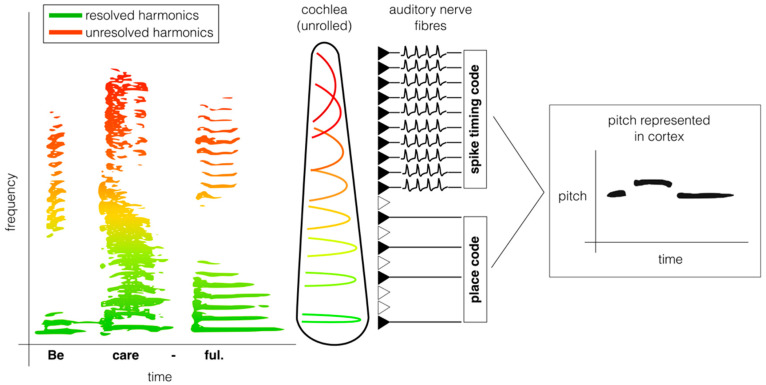
**Resolved and unresolved harmonics can each give rise to pitch perception through distinct neural mechanisms.** Most natural sounds, such as speech, contain both resolved (green) and unresolved (red) harmonics (**left plot**). On a linear scale, frequency receptive fields in the cochlea (i.e., “cochlear filters”) are wider for higher frequencies. As a result, low-numbered harmonics are “resolved” in the cochlea, with only one harmonic falling within the receptive field of a given neuron. Auditory nerve fibres tuned to higher-numbered harmonics instead respond to multiple harmonics within their frequency receptive field, so these harmonics are termed “unresolved”. The resolved harmonics produce a place code representation of F0 in the auditory nerve (**middle plot**). The F0 of unresolved harmonics are instead encoded in an explicit spike timing code, as the auditory nerve fibres phase lock to their summed periodicity at F0. Neurons in the auditory cortex may combine these two codes to provide a cue-invariant representation of pitch (**right plot**).

At least two acoustic characteristics determine a sound’s pitch. First, the waveform is periodic with a repetition rate of F0, enabling the auditory nerve to synchronise its spikes to this timing cue (“phase locking”). Second, the frequency components of the sound are all harmonics of a common F0, and the spacing of these harmonics is represented as a place code across the tonotopic map (Figure 2). The place code of F0 is only *resolved* for lower harmonics (the first 5–8 harmonics in human listeners). The frequency bandwidths of neurons tuned to higher harmonics are broader on a linear frequency scale, including neighbouring harmonics, so these are *unresolved* [57]. Human psychophysical studies suggest that both resolved harmonics and temporal envelopes contribute to pitch perception, but resolved harmonics provide finer pitch acuity [20,58,59,60]. Other mammals also show evidence of this dual pitch extraction strategy [10,11,12,45,61,62], but non-humans often rely more on temporal pitch due to poorer frequency resolution in their cochlea [12,45,61].

Both resolved and unresolved harmonics can support sound segregation in streaming tasks. Human listeners show similar stream build-up and oddball detection in the attended stream using either of these pitch cues [63,64], but pitch-based streaming effects in humans tend to be stronger when harmonic tones contain more resolved harmonics, and weaker when they rely more on unresolved harmonics [65,66]. In contrast, Madsen et al. [67] found no effect of harmonic resolvability on a multi-talker listening task. However, their process of low- and high-pass filtering speech to isolate harmonics disrupted formant cues that are critical to intelligibility and thus task performance. This can be minimised in future work by manipulating only harmonic components of speech, leaving noise-filled formant structures intact [38].

The variability in the number of resolved harmonics available across species offers a comparative framework for understanding how pitch is utilised for selective listening. For example, we might predict that ferrets and rodents might segregate sounds more effectively with unresolved harmonics, in contrast to human listeners. To date, the role of different pitch cues in selective listening performance remains unexplored in animal behaviour. Neurophysiological studies in animals, meanwhile, have provided insights into how these two F0 cues are represented in neural spiking responses. A subset of neurons in the auditory cortex of marmosets and ferrets are specialised to process resolved harmonic F0 cues, while others extract the periodicity of F0 from unresolved harmonics [11,68]. At a population level, neurons in macaque primary auditory cortex can represent resolved harmonic patterns as a place code, while low F0s are also represented by neuronal phase-locking to F0 [69].

When two-tone streaming stimuli are presented to animals, auditory cortical neurons show more distinct spiking responses to A and B sounds when their F0 difference is larger, even for unresolved harmonic complex tones that only differ in their temporal periodicity [70,71]. This provides a neural correlate of temporal-pitch-based stream segregation of the same sequences by human listeners [72,73]. Furthermore, the differences in the neural response to A and B complexes developed over the first 5–20 s of sequence presentation, mirroring the time frame of streaming build-up in human perception [71]. In this manner, streaming build-up provides an objective of streaming in animal models. These findings are from passively listening animals, leaving the link between neural activity and attentional effects unconfirmed. However, the fact that neural segregation occurs without active task engagement suggests that individual pitch cues, such as the temporal envelopes of unresolved harmonics, can support the binding of auditory objects in these species.

## 4. Neural Activity Supporting Pitch-Based Selective Listening

Selective listening requires coordinated activity across widely distributed brain areas within and outside the auditory system. Even the neural basis for perceiving the pitch of an isolated sound source is poorly understood; the auditory cortex is held to play a key role in pitch perception, but it remains unclear if pitch is processed within a specialised pitch centre or through representations spread across multiple auditory cortical regions [14,74]. Understanding the more widespread neural processes that integrate the bottom-up binding of pitch features with top-down attentional selection is an even more daunting challenge. Non-invasive methods commonly used to study human brain function (EEG, MEG, and fMRI) and, more recently, ECoG in epilepsy patients, are ideally suited to sample such broad neural activation patterns.

EEG combines broad spatial sampling of neural activity with fast temporal fidelity, and has provided physiological evidence of selective listening being a two-stage process. Studies using concurrent vowels or mistuned harmonics as stimuli have observed an early object-related negativity, thought to reflect bottom-up feature binding, when both sounds are presented at sufficiently different pitches, even if the subject is not attending to them [75,76,77]. A later positive (P400) event-related potential follows only if the participant is actively listening to the sounds to perform a behavioural task, and may therefore result from top-down attention. Functional MRI, which can better localise the source of neural activity to particular brain regions, has shown that activity in left auditory thalamus and auditory cortex (including Heschl’s gyrus, planum temporale, and the superior temporal gyrus) is increased when listeners segregate two vowels, harmonic tones, or speech segments presented simultaneously at different pitches [78,79].

Human electrophysiological approaches have also been applied to more naturalistic multi-talker tasks, where listeners are asked to attend to one voice and ignore another. A wealth of EEG studies has shown that attention selectively enhances the neural representation of the target speech envelope [80,81,82,83,84]. However, these studies have usually presented the two speakers lateralised to different sides of the head, so segregation will largely result from spatial cues rather than pitch. A smaller number of investigations have instead presented a male and female voice diotically, where pitch cues will dominate segregation, and these also show enhanced phase-locked cortical tracking of the target speech envelope, with weaker neural signatures of the ignored speech [85,86]. ECoG recordings in epilepsy patients have provided further insights into the cortical distribution of selective listening effects. While neural activity in primary auditory cortex represents both voices in a multi-talker listening task, even when selectively listening to one voice, higher auditory cortical areas dynamically track the attended speaker more exclusively [6,24]. The spectrogram of the attended, but not ignored, voice can be reconstructed from these higher cortical responses [87]. Together, these studies suggest that selective attention enhances the representation of the attended talker, particularly in the secondary auditory cortex. However, a limitation of the male/female talker design is that they do not carefully isolate or parameterize pitch cues (F0); timbre (i.e., different voice quality present in higher formants of the sound spectrum) may also be used to segregate voices in these experiments. Studies that present the same voice at a range of different pitches would better examine the specific role of pitch in multi-talker segregation.

Two-tone streaming sequences have also been used to identify neural markers of auditory scene analysis, where the role of pitch in segregation has been better controlled. Early EEG studies using this paradigm have shown increased positive evoked responses [88] or mismatched negativity responses [89] to pure tone sequences when they are perceptually segregated into two streams. Functional MRI has further shown that the increased response to segregated pure tone streams is localised in the auditory cortex [90] (but note that Cusack [91] reports selective activation only in higher regions of the intraparietal sulcus). At least one study has shown that the neurobiological basis of streaming the frequency of pure tones may extend to the pitch of spectrally overlapping harmonic tone complexes: Gutschalk et al. [92] employed fMRI and MEG to show increased primary and secondary auditory cortical activation when listeners perceived two streams based on F0 separation. Together, the above studies suggest that the human auditory cortex, particularly non-primary regions, may play a key role in frequency- and pitch-based selective listening.

Due to the challenges of training animals on selective listening tasks (discussed above), much of the work examining neural correlates of sound source segregation has been carried out in passively listening or anaesthetised animals. The pitch of two simultaneously presented harmonic sounds are represented with a combination of tonotopic place codes (for resolved harmonics) and envelope-locked temporal spiking patterns as early as the auditory nerve [93,94,95,96]. The F0s of spectrally overlapping sounds continue to be represented at subcortical nuclei in the auditory system, including the ventral cochlear nucleus [97], and therefore retain the information needed for later pitch-based segregation in the cortex. At the level of the primary auditory cortex, the F0s of each harmonic sound in the pair are represented as distinct rate-place codes across populations of neurons [69,98]. There has not been a clear lateralization of pitch processing in animal models, in contrast to the left hemisphere dominance often reported in human auditory cortex [78,99]. Studies have not yet tested if these neural correlates in animals can drive selective attention in these species, but this remains a goal of future investigations in awake, behaving animals.

Based on the human EEG and ECoG studies above, we would expect that responses in auditory cortical neurons would become dominated by representations of the target sound as animals engage attention. Many studies have demonstrated that the auditory cortex provides a stable representation of dynamic sound “foregrounds” in the presence of stationary “background” noise, even in passively listening or anaesthetised animals [100,101,102,103,104,105,106], but these neural mechanisms may be less relevant to segregating two dynamic sounds that differ only in pitch. Noise vocoding, which degrades pitch information, reduced the decoding performance for vocalisations presented in a noise background throughout the auditory subcortical and cortical pathway [106], which may suggest that pitch cues at least partially contribute to the noise invariance observed in the above studies. However, F0 effects on segregation need to be more directly investigated while controlling for spectral timbre and slower temporal modulation cues.

While two-tone streaming paradigms have rarely been trained in animals, the neural correlates of ABA- or AB pure tone sequences that differ in frequency have been extensively studied in passively listening animals, including microelectrode recordings in macaques [107,108,109], ferrets [110], bats [111], rats [112], guinea pigs [113], and songbirds [51,114]. These studies have corroborated and extended results in human neurophysiological work at the single neuron level. In these paradigms, the B tone elicits reduced spiking responses when A and B are close in frequency, mirroring the perceptual fusion observed in human streaming. Segregation is decodable in individual neurons, but population-level signals align well with the perceptual boundary between integration and segregation observed in human listeners. This suggests that population dynamics may be more relevant for perceptual organisation than the output of any one neuron. Studying these population codes is technically challenging, particularly for pitch-based selective listening, because pitch representations are distributed widely across the auditory cortex in most mammals [69,115,116,117,118,119,120]. Computational models that decode auditory objects from distributed pitch representations could offer testable predictions for the neural mechanisms of pitch-based selective listening. Streaming studies that employ harmonic, spectrally overlapping stimuli and in which the animal is actively listening are also required to understand how selective attention manifests at a single neuron level.

Together, the human and animal neurophysiological studies point to a key role of the auditory cortex in pitch-guided selective listening, within a hierarchical network that employs bottom-up representations of pitch for perceptual binding and top-down attention through selective modulation of a segregated target.

## 5. Does Attention Enhance the Target or Suppress the Distractor?

A key mechanistic question is whether selective listening acts primarily through *target enhancement* (increasing the gain of the harmonic target representation in auditory cortex), *masker suppression* (inhibiting representations of a competing harmonic background sound), or both. This question was addressed in concurrent vowel identification tasks in humans, which found improved target recognition when the masking vowel was harmonic rather than inharmonic [121,122,123]. This led to the “*harmonic cancellation*” hypothesis, which proposes that harmonic maskers are grouped and removed from the mixture to better reveal the target [124].

More recent studies have challenged the harmonic cancellation hypothesis. In a speech-in-noise task, Steinmetzger and Rosen [125] created inharmonic maskers by frequency-shifting all spectral components equally, thereby preserving spectral regularity while disrupting harmonicity. They found no difference in target speech intelligibility between harmonic and inharmonic maskers, whether the masker F0 was static or dynamic. They argued that the benefits of harmonic maskers in earlier concurrent vowel experiments may have resulted from opportunities for spectral glimpsing and reduced temporal envelope fluctuations in harmonic maskers, rather than masker harmonicity per se. Taken together, human psychophysical data suggest that any advantage of masker harmonicity is context-dependent, making it difficult to draw conclusions about the relative importance of target and maskers on attentional modulation. It is worth noting that in real-world listening environments, one is often faced with segregating multiple harmonic sources, but not the frequency-shifted stimuli employed by Steinmetzger and Rosen [125].

Disentangling the contributions of excitation and suppression in the human brain remains difficult, even with modern neurophysiological approaches, whereas techniques employed in animal models allow cellular and circuit-level investigations of these processes. Neural responses could be compared between excitatory and inhibitory neurons in the auditory cortex, and causality can be tested by manipulating activity of targeted neuronal types. The potential of this approach is illustrated by previous studies of auditory attention outside the context of pitch-based listening. For example, for zebra finches detecting a target song in a background of bird chorus, broad-spiking (putative excitatory), but not narrow-spiking (putative inhibitory), neurons in the higher auditory cortex encoded target songs in a noise-robust manner [22]. This study used pharmacological approaches to further demonstrate that target selectivity arises from GABA-dependent forward suppression. In mice, inhibiting parvalbumin-positive interneurons in the auditory cortex degraded cortical representations of dynamic sounds in static broadband noise [126]. These studies show how inhibitory circuits can shape foreground-background coding in auditory cortex. Future work should leverage these approaches to test whether inhibitory mechanisms preferentially mediate masker suppression, while excitatory gain and top-down feedback preferentially drive target enhancement, and if harmonicity can guide these processes.

## 6. Conclusions

**Figure 3:**
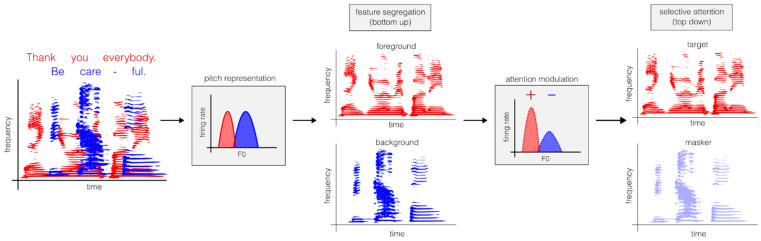
Selective listening conceptualised as a two-stage task requiring bottom-up feature segregation and top-down selective attention. A complex acoustic scene with multiple sound sources, such as two people’s voices (red and blue), must first be encoded and relayed from peripheral to cortical auditory stations. Along this ascending pathway, each voice can be segregated by binding harmonic components of its F0. Higher cortical areas, potentially outside the auditory system, may enhance the auditory cortical representation of the target speaker (and/or inhibit the representation of the ignored speaker) through top-down modulation.

The existence of a common fundamental frequency in natural harmonic sounds allows their frequency components to be easily bound together into a single auditory object. These bound feature representations can then be targeted by attentional systems to enhance neural representations of a target object (Figure 3). Our perception of pitch thus provides a valuable cue for selective listening. Much of the above work has focused on speech perception, but pitch also aids the segregation of instruments in music [127]. Beyond the human soundscapes of speech and music, pitch plays a more fundamental, cross-species role in binding and segregating a wide range of naturally harmonic sound sources, from birdsong to the whistling of wind.

Sensorineural hearing loss commonly occurs with ageing and is associated with poorer pitch discrimination [128,129] as well as difficulty using pitch cues in multi-talker listening tasks [2,74,130]. Pitch perception is even more severely degraded in cochlear implant users [128]. As a result, deficits in pitch processing are likely to contribute to challenges that people with hearing impairments experience when following conversations in noisy environments [131]. It may also play a role in their elevated risk of dementia [132], highlighting the clinical significance of research in this area.

A host of paradigms have been employed to demonstrate how humans and other animals can direct their attention to pitch cues (Table 1). While cortical correlates of pitch-based selective attention have been described, many fundamental questions remain about these neurobiological mechanisms (Box 1). For example, while attention is known to selectively enhance target representations in auditory cortex, it remains unclear how these representations are anatomically targeted by attentional brain regions that lack acoustic details. Furthermore, does attentional modulation involve enhancement of target representations, suppression of background sounds, or a combination of both? We propose that a better understanding of pitch-based selective listening across species can be realised by aligning three levels of analysis: (i) controlled behavioural paradigms that isolate pitch cues, and are not language-specific; (ii) neural recordings and manipulations that isolate neural circuits and cell types; and (iii) computational models that propose how attentional modulation can target and modify representations of a spectrally complex target sound in the auditory cortex.

**Table 1:** Comparative paradigms used to study pitch-based segregation across humans and non-human animals, with selected citations.

Paradigm/ Stimulus	Species	Behavioural Findings	Neurophysiological Findings
Concurrent vowels	Human	F0 differences improve reporting both vowels [29,30,31,32,121,123].	Increased activation in auditory cortex when both vowels are successfully identified [75,78].
	Non-human animals		Neural encoding of both vowels in auditory nerve population using place and temporal codes. Population code for both vowels in place code in auditory cortex [98].
Mistuned harmonics	Human	Detection of mistuned harmonics leads to perceptual segregation of the mistuned component as a separate object [33,34,35,36].	EEG/MEG show distinct responses to mistuned vs. harmonic tones; fMRI implicates auditory cortex in detecting harmonic violations [76].
	Non- human animals	Animals (ferrets, gerbils, birds) detect mistuned harmonics, demonstrating perceptual grouping based on harmonicity [43,44,45,46].	Auditory cortical and subcortical neurons differentiate harmonic from mistuned tones, reflecting harmonicity-based segregation mechanisms [43,133].
Two-tone streaming paradigms (pure tones or harmonic complexes)	Human	Complex F0 or tone frequency separation drives perceptual segregation; small differences (<10%) yield fusion (“gallop”), larger separations yield two streams [1,41,42].	EEG shows early negativity for automatic feature binding, and later positivity (P400) with active attention. fMRI/MEG shows increased auditory cortical responses for segregated vs. fused streams [88,89,90,91,92].
	Non-human animals	Monkeys, ferrets, birds, and even fish segregate tone streams when large enough frequency difference. Lack of studies of complex F0 streaming [47,48,49,50].	Populations of auditory cortical neurons show more distinct responses to alternating tones when presented with a larger frequency difference. Similar effects for the temporal pitch differences in harmonic complexes [51,70,71,107,108,109,110,111,112,113,114].
Multi-talker speech	Human	Female/male voice differences (high/low F0, respectively) facilitate segregation and speech intelligibility [27,28,38].	EEG and ECoG: enhanced cortical tracking of attended speech envelope. MEG/fMRI: selective enhancement of target voice in secondary auditory cortex [6,24,79,85,86,87].

Box 1 **Open research questions and suggested experimental directions.**

Open research questions and future directions

**Where and how is pitch represented across the auditory hierarchy?**

Is there a specialised “pitch centre” or distributed coding across auditory cortex? Multi-scale recordings and causal manipulations in animal models are required to help resolve this.

**How does attention interact with pitch representations?**

Does selective attention act primarily by enhancing target-related activity, suppressing distractors, or both?

**What is the relative importance of resolved versus temporal pitch cues?**

Does attention modulate these F0 cues independently, or does it act on pitch representations after these have been combined?

**How does pitch-based segregation generalise from pure tones to complex sounds?**

Most studies use simplified tones which can be trivially segregated via frequency place codes. Dynamic, complex harmonic stimuli with competing sources are needed to test whether tone-based mechanisms generalise to real-world listening.

**How well does pitch support selective listening in multi-talker tasks?**

There is a wealth of behavioural and neurophysiological studies examining selective attention to speech in humans, but almost none of these isolate pitch cues (F0) from other potential segregation cues, such as timbre and sound source location.

**How does hearing loss reshape pitch-based selective attention?**

Peripheral deficits alter harmonic coding, but how central auditory circuits adapt (or fail to adapt) remains unclear.

**How can animal–human comparisons be more tightly aligned?**

Parallel paradigms using harmonic complexes and streaming tasks across species will clarify which mechanisms are evolutionarily conserved and which are species-specific.

## Data Availability

No new data were created or analyzed in this study.

## Abbreviations

dB	decibels
EEG	electroencephalography
ECoG	electrocorticography
fMRI	functional magnetic resonance imaging
F0	fundamental frequency
Hz	Hertz
MEG	magnetoencephalography
